# Supplementary material for: Chronic Kidney Disease Diets for Kidney Failure Prevention: Insights from the IL-11 Paradigm
Source: Nutrients. 2024 Apr 29;16(9):1342. doi: 10.3390/nu16091342 (PMC11085624; doi:10.3390/nu16091342)
Supplement: Supplementary file 1 [file nutrients-16-01342-s001.zip › nutrients-2953842-supplementary.pdf]

Supplementary Materials
Content:

1. **Abbreviations in the Tables**
2. **Legend for nutrients used in the Figures.**
3. **Table S1**
4. **Table S2**
5. **Table S3**

1. Abbreviations in the Tables

Statistics:
n, number of participants who completed study;
NI, not indicated;
NSD, no significant difference;
C, Control
M, Men/Male =; Women/Female = F

Assessments:
CRP: C-Reactive Protein
eGFR: Estimated Glomerular Filtration Rate
HDL: High Density Lipoprotein
LDL: Low Density Lipoprotein
PDS: Plant-Diet Score used to assess adherence to a plant-based diet, where higher scores indicate a higher proportion of plant-based foods in one's diet
TG: Triglycerides
QOL, Quality of Life
UC: Usual Care
UACR: Urine Albumin to Creatinine Ratio
UP: Urinary Protein

Diets-related:
ALA: α-Linolenic Acid
DASH: Dietary Approach to Stop Hypertension
DHA: Docosahexaenoic Acid
EPA: Eicosapentaenoic Acid
erMedDiet: Energy Reduced Mediterranean Diet
FV: Fruit/Vegetable Diet
HDP: Health Dietary Pattern
HEI: Healthy Eating Index
LFD: Low-Fat Diet
mKIDMED: Mediterranean Diet for Children and Young Adults
OO: Olive Oil
SD: Standard Therapeutic Diet (usually Low-Protein Diet for CKD)
SD/UD: Standard/Usual Diet
tMedDiet: Trichopoulou Mediterranean Diet
UD: Usual Diet (average diet for individuals in the country of study)

Diseases:
CKD: Chronic Kidney Disease
CVD: Cardiovascular Disease
DM: Diabetes
DLD: Dyslipidemia
ESRD: End-Stage Renal Disease

HD: Hemodialysis
HTN: Hypertension
MetS: Metabolic Syndrome
PD: Peritoneal Dialysis
T1DM: Type 1 Diabetes
T2DM: Type 2 Diabetes

2. Legend for nutrients used in the Figures.

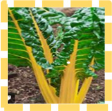

Golden Swiss chard source for lutein and zeaxanthin and with lower content of oxalates

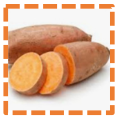

Sweet potato source for provitamin A

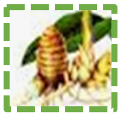

Turmeric source for curcumin

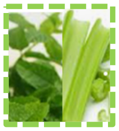

Mint and celery source for terpenoid β-elemene

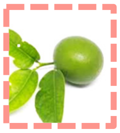

Lime (*Rutaceae* families) **source** for a coumarin derivative osthole and quercetin

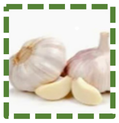

Garlic source for sulfur-containing compound allicin

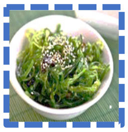

Algae source for ω3 fatty acids DHA and EPA

Table S1. Clinical Trials Involving Dietary Patterns, Foods, and Nutrients That Might Prevent CKD Development in Populations at Risk.
Diet adherence or in protein diet score from baseline to 1 year in 4 groups: 1) Decrease/ Maintain, 2) T1, Lowest tertile increase/adherence, 3) T2, Middle tertile increase/adherence, 4) T3, Highest tertile increase/adherence.

Diet	Outcome	Duration	Baseline CKD Stage & Non-CKD Conditions/Criteria	Source
MedDiet/OO (n=214)<br><br>MedDiet/nuts (n=227)<br><br>LFD Control (n=224)	All improved kidney function, ↑ eGFR<br><br>NSD in MedDiet vs LFD	1 y	No CKD<br><br>CVD risk but no CVD	{Diaz-Lopez, 2012 #91}

MedDiet/OO (n=1,282)<br><br>MedDiet/nuts (n=1,142)<br><br>LFD (C) (n=1,190)	64 cases of DM nephropathy, NSD<br><br>51 cases of DM nephropathy, NSD<br><br>53 cases of DM nephropathy, NSD	6 y	No CKD<br><br>T2DM, CVD risk but no CVD	{Diaz-Lopez, 2015 #92}
erMedDiet, T3* (n=1,423 of 5,675)<br><br>TMedDiet, T3* (n=534 of 5,675)<br><br>DASH, T3* (n=852 of 5,675)<br><br>PDS, T3* (n= 98 of 5,675)	↓ risk eGFR decline, larger ↑ eGFR<br><br>NSD renal outcomes<br><br>NSD renal outcomes<br><br>↑ risk eGFR decline, larger ↓ eGFR	1 y	No CKD<br><br>MetS, BMI of 27-40 kg/m2, no CVD, no extreme high/low caloric intake.	{Valle-Hita, 2022 #95}
DASH T3* highest adherence vs T1 lowest adherence (n=14,882)<br><br>Red meat, processed meat, and protein, Q5 high intake vs Q1 low intake (n=14,882)<br><br>Nuts, legumes, LF dairy, magnesium, and calcium, Q5 high intake vs Q1 low intake. (n=14,882)	↓ CKD incidence<br><br>↑ CKD incidence<br><br>↓ CKD incidence	25 y	No CKD<br><br>Selected communities, majority African- American or Caucasian	{Rebholz, 2016 #93}
DASH T3 highest adherence vs T1 lowest adherence (n=584 of 2,058)<br><br>*n=869 with poverty*<br><br>*n= 1,189 non-poverty*	↑ CKD risk only significant in poverty group<br><br>CKD in 5.6% of participants with poverty<br><br>No CKD in 3.8% of participants without poverty	Non-temporal data analysis	Urban community, 42% with poverty	{Crews, 2015 #100}
mKIDMED, higher scores (n=461)	NSD of developing microalbuminuria	Varies,	No CKD<br><br>Youth onset T1DM.	{Costacou, 2018 #94}

DASH, higher scores (n=462)<br><br>HEI (n=462)	NSD of developing microalbuminuria<br><br>↓ risk of developing microalbuminuria (borderline significance)	≥5 y. Baseline to follow- up visits	Age < 18 years at start of study.	
DASH 18% protein (n=127)<br><br>FV 15% protein (n=124)<br><br>UD 15% protein (C) (n=120)	NSD *(Lack of ↑ AER despite 3% more protein kcal suggests it may be protective).*<br><br>↓ urinary albumin excretion rate (AER) only when baseline is higher than normal AER.	8 wks	No CKD<br><br>Pre-hypertension or stage 1 hypertension. No DM	{Jacobs, 2009 #102}
FV (Based on serum carotenoid biomarker for FV diet) (n=2,152)	↓ incidence of rapid kidney decline (> 15% eGFR decline in 5 yrs)<br><br>*Insufficient cases of incident CKD at year 20 for analysis*	5 y<br><br>(eGFR data from years 15 & 20)	No CKD<br><br>Serum carotenoid at year 15 of study	{Hirahatake, 2019 #96}
Meta-analysis of HDP* (n=630,108 across 18 studies)	↓ incidence of CKD & microalbuminuria<br><br>NSD eGFR decline	2-23 y	No CKD<br><br>N/A	{Bach, 2019 #98}
Meta-analysis of HDP* & light-moderate alcohol intake (n=149,958 across 17 studies)	↓ risk of CKD HDP	NI	No CKD<br><br>N/A	{He, 2021 #99}
Omega-3 400 mg/ day EPA/ DHA in margarine (n=576)<br><br>Omega-3 2 g/day ALA in margarine (n=601)<br><br>Omega-3 400 mg/ day EPA/ DHA / 2 g/ day ALA in margarine (n=574)<br>Margarine placebo (n=593)	Long-term EPA/ DHA supplements:<br>Small benefits for kidney function with slower annual decline in eGFR.<br>More significant benefits in patients with CKD than without.<br><br>NSD in incident CKD or rapid kidney function decline.<br><br>ALA supplement: NSD in kidney function	40 mo	Early or no CKD<br><br>History of myocardial infarction, at risk for CKD, mean eGFR 78.5 mL/ min).	{Hoogeveen, 2014 #175}

Meta- analysis of Omega 3 FA (seafood or plants) (n=25,570 across 19 studies)	↑ Omega 3 from seafood: ↓ risk of CKD & slower annual decline in eGFR.<br><br>↑ Omega 3 from plants: NSD	Median 11.3 hrs	No CKD<br><br>N/A (eGFR > 60 mL/min).	{Ong, 2023 #176}
Extra virgin OO with high oleic acid and phenolic content. (n=29)<br><br>Refined OO with low oleic acid and phenolic content. (n=34)	Both showed improved coronary artery disease biomarkers. No change CKD, CVD, or DM biomarkers.<br><br>*NSD high vs. low phenolic OO*	6 wks	No CKD<br><br>Non. Self-reported, healthy.	{Silva, 2015 #101}

Table S2. Clinical Trials Involving Studies of Dietary Patterns, Foods, and Nutrients That Might Prevent CKD Progression.

Diet	Outcome	Duration	Baseline CKD Stage, eGFR, Albuminuria, or Proteinuria	Other Criteria	Source
MedDiet:<br>LPD for ≥ 6 mo (baseline),<br><br>then IMD for 14 d, (n=40)<br><br>then IMOD for 14d (n=40, same as prior)	IMD & IMOD:<br>↓ hyperphosphatemia,<br>↓ BMI<br>↓ DLD<br>↓ CVD risk<br><br>IMOD additional observation:<br>↑ lean muscle mass<br>↓ albuminuria.	28 d	Stage 2-3, NI	Only chronic glomerulonephritis	{Di Daniele, 2014 #222}
MedDiet (after LFD baseline)<br>(n=21)<br><br>Continue LFD (C)<br>(n=16)	↓ DLD<br>↓ Oxidative stress<br>↓ CVD risk	6 mo	ESRD, NI, NI	Renal transplant, stable	{Stachowska, 2005 #126}
MedDiet<br>(n=20)<br><br>SD (C)<br>(n=20)	↑ food consumption, improved CVD and nutritional states<br><br>↓ DLD and inflammation	90 d	Stage G2	DLD, no DM	{Mekki, 2010 #224}
NNRD for 1 wk, washout for 1-3 wks<br>(n=18)<br><br>UD (C) for 1 wk (or vice versa)<br>(n=18 same as prior)	↓ metabolic acidosis, urinary excretion of uremic toxins, indoxyl sulfate, & p-cresyl sulfate	3-6 wks	Stage 3b-4, NI	Stable CKD	{Hansen, 2023 #115}
NNRD for 1 wk, washout for 1-3 wks<br>(n=18)<br><br>UD (C) for 1 wk (or vice versa)<br>(n=18 same as prior)	↓ urinary excretion of phosphorus, urea nitrogen<br>↓ plasma intact FGF23, normalized plasma phosphorus<br><br>*NNRD is well tolerated*	3-6 wks	Stage 3b-4, NI	Stable CKD	{Salomo, 2019 #122}
Vegetarian SVLPD<br>(n=27)<br><br>SD (C)<br>(n=26)	↓ serum urea, improved acid-base, improved calcium-phosphorus metabolism, stable eGFR<br><br>↓ initiation of renal replacement therapy, maintained nutritional status	48 wks	Stage 4-5, NI	Non-diabetic, well-controlled BP, stable CKD	{Mircescu, 2007 #107}
FV dosed to decrease dietary acid by half.	Improved metabolic acidosis, stable eGFR	5 y	Stage 3, UACR > 200 mg/g	HTN, no DM, CKD with	{Goraya, 2019 #112}

(n=36)<br><br>Oral Sodium Bicarbonate (n=36)<br><br>UC (C) (n=36)	+<br>Improved CVD risk factors, including<br>↓ systolic BP<br>↓ LDL<br>↓ BMI<br>↑ serum Vitamin K<br><br>Improved metabolic acidosis, stable eGFR			macroalbuminuria minuria.<br>Metabolic acidosis (PTCO2 = 22-24 mM)	
FV dosed to decrease dietary acid by half (n=36)<br><br>Oral Sodium Bicarbonate (n=35)	Improved metabolic acidosis, stable eGFR,<br>↓ urine indices of kidney injury<br>+<br>↓ systolic BP and BMI<br>*Note: No ↑ risk for hyperkalemia but ↑ potassium urinary excretion, so potential hyperkalemia if low eGFR or other risk factors.*<br><br>Improved metabolic acidosis, stable eGFR and ↓ urine indices of kidney injury	1 y	Stage 4, NI	HTN, no DM.<br>Metabolic acidosis (PTCO2 < 22 mM)	{Goraya, 2013 #113}
FV dosed to decrease dietary acid by half (n=26 CKD1, n=40 CKD2)<br><br>Oral Sodium Bicarbonate (n=26 CKD1, n=40 CKD2)<br><br>Time Control (n=27 CKD1, n=40 CKD2)	↓ urine indices of kidney injury & stable eGFR in CKD2 but not in CKD1.<br>+<br>↓ systolic BP & ↓ BMI in both CKD1 & CKD2.<br><br>↓ urine indices of kidney injury & stable eGFR in CKD2 but not in CKD1.	30 d	Stage 1-2, uACR > 200 mg/g	Early CKD due to HTN with macroalbuminuria. No DM or metabolic acidosis (PTCO2 ≥ 24.5 mM).	{Goraya, 2012 #114}
Soy protein (35% soy, 35% animal, 30% vegetable) (n=20)<br><br>Animal protein (C) (70% animal, 30% vegetable) (n=21)	↓ urinary creatinine<br>↓ urea nitrogen, but no change in eGFR.<br><br>Improved lipid profile,<br>↓ glucose, but no change in BP.	4 y	Stage 1-2, NI, UP mean 513 mg/day	Diabetic nephro-pathy, T2DM, proteinuria, HTN.	{Azadbakht, 2008 #110}

Soy LPD for 6 months<br>(n=9)<br><br>then Standard Diet for 6 months (or vice versa)<br>(n=9 same as prior)	↓ rate CKD progression & similar nutrition status.<br>+<br>↓ BUN, urine urea nitrogen, protein catabolic rate, 24-h urine creatinine & phosphate.<br>↓ protein & phosphate intake<br>↑ caloric intake<br><br>↓ rate CKD progression & similar nutrition status.	1 y	Stage G3a-4, UP mean 1.2 g/day.	N/A	{Soroka, 1998 #106}
Soy-based protein for 8 wks,<br>(n=12)<br><br>then animal-based protein for 8 wks (or vice versa)<br>(n=12, same as prior)	↓ eGFR (↓ hyperfiltration) & improved lipid profile.	20 wks	Stage 1, > 120, UP > 300 mg/day	T1DM diabetic nephropathy with hyperfiltration. Good glycemic control.	{Stephenson, 2005 #111}
Soy-based protein supplement<br>(n=15)<br><br>Milk-based protein supplement (C)<br>(n=10)	Individuals:<br>↓ markers of inflammation,<br>↑ markers of nutrition<br><br>Group level:<br>NSD in inflammation or nutrition	8 wks	ESRD, NI, NI	HD > 4 mo. ≥ marker of inflammation	{Fanti, 2006 #121}
Soy protein<br>(n=18)<br><br>UD, no soy (C)<br>(n=18)	↓ CVD risk with<br>↓ Lp(a).<br><br>NSD in TC, LDL, HDL, or TG.	8 wks	ESRD, NI, NI	PD	{Tabibi, 2010 #132}
Soy protein<br>(n=18)<br><br>UD, no soy (C)<br>(n=18)	↓ thrombosis risk with<br>↓ plasma coagulation factor IX.<br><br>NSD ox-LDL, fibrinogen, or coagulation factors VII and X.	8 wks	ESRD, NI, NI	PD	{Imani, 2009 #133}
Cereal, cookies, and bars without added fiber (C) for 2 wks,<br>(n=15)<br><br>then similar food with added fiber (23 g/day) for 4 wks<br>(n=15, same as prior)	↓ constipation with ↑ stool frequency, improved lipid profile, & no change in overall QOL.	6 wks	Stage 3-5, NI	N/A	{Salmean, 2013 #125}

Freshly squeezed pomegranate juice (PJ) for 8 weeks, (n=41)<br><br>then washout for 4 weeks, and UC for 8 weeks (or vice versa) (n=41, same as prior)	↓ BP, triglycerides.<br>↑ HDL.<br><br>Signs of ↓ oxidative stress (e.g., ↑ total antioxidant capacity) & ↓ inflammation (e.g., ↓ IL-6).	20 wks	ESRD, NI, NI	HD ≥ 3 mo	{Barati Boldaji, 2020 #128}
Fish oil (4.6 g n-3) supplement (n=14)<br><br>OO supplement (n=15)	Both:<br>↑ fibrinolytic potential after 6 months. No change in transcapillary escape rate of albumin or procoagulant activity.	1 y	NI, NI, UA > 300 mg/day	Diabetic nephropathy with albuminuria. Insulin- dependent DM.	{Myrup, 2001 #135}
MedDiet, T3* (n=2,403)<br><br>DASH, T3* (n=2,403 same as prior)<br><br>HEI 2015, T3* (n=2,403, same as prior)<br><br>Alternative HEI 2010* (n=2,403 same as prior)<br><br>Intake of vegetables & nuts in MedDiet, T3* (n=2,403, same as prior)	MedDiet, DASH, HEI 2015, HEI 2010: ↓ risk of CKD progression¥, and risk of all-cause mortality<br><br>¥ Defined as eGFR decline > 50% from baseline or ESRD	Max 14 y	Stage 2-4, Various	N/A	{Hu, 2021 #108}
Overall diet quality (n = 68, n = 40 in optional fecal sub-study)<br><br>Fiber intake, highest scores (n = 68)<br><br>Healthy plant-based diet index (hPDI), highest scores (n = 68)	Affects microbiome & production of uremic toxins<br><br>↓ total indoxyl sulfate<br><br>↓ free p-cresyl sulfate	Non-temporal data analysis	Stage 3-4, NI	Stable CKD	{McFarlane, 2022 #124}
Meta-analysis of HDP*<br><br>(n=15,285 across 7 studies)	↓ risk of mortality<br><br>NSD risk of ESRD	≥ 24 wks	Stage 2-5, NI	One study eGFR 20-70 mL/min, and one study ESRD HD.	{Kelly, 2017 #136}

Plant-Based Protein, Q5*<br>(n=588 of 2,938)<br><br>**Q5 indicates highest % plant protein*	↓ urinary creatinine, urea nitrogen, but no change in eGFR.<br>Improved lipid profile, ↓ glucose, but no change in BP.	Non-temporal data analysis	Stage 2-4, NI	N/A	{Scialla, 2012 #117}

Supplementary Table S3. Clinical Trials Involving Studies of Other Nutrients That Might Prevent CKD Progression.

Nutrient	Outcome	Duration	Baseline CKD Stage & Non-CKD Conditions/Criteria	Source
VLCKD<br>(n=92)<br><br>Note: Meal replacement protein sources are whey and plant- based and never > 1.5 g/kg IBW/d	Safe and effective weight loss, significant proportion of stage G2 CKD resolved to normal eGFR (Stage G2),<br><br>↓ BMI, TG, & improved metabolic profile	3 mo	Stage 2<br><br>Obesity and early (Stage G2)<br>(n=38)<br><br>Obesity and no CKD (Stage G1, control).<br>(n=54)	{Bruci, 2020 #268}
VLCKD and exercise<br>(n=6*)<br><br>*1 participant completed only 7 weeks but was still included in analyses	Improved DM status, 36% reduction in albuminuria but not significant, ↓ creatinine and BMI	12 wks	Stage 3-4<br><br>Advanced diabetic nephropathy with obesity, non-dialysis (eGFR < 40 mL/ min and UA > 30 mg/d) DM	{Friedman, 2013 #269}
Resveratrol 500 mg supplement for 4 weeks, washout for 8 weeks, oral placebo for 4 weeks or vice versa<br>(n=20)	NSD in uremic toxins produced by gut microbiota	16 wks	Stage 3-4<br><br>Non-dialysis with eGFR < 60 mL/min. Low protein diet at least 6 mo before intervention.	{Alvarenga, 2022 #263}
Resveratrol 500 mg supplement for 4 weeks, washout for 8 weeks, oral placebo for 4 weeks or vice versa<br>(n=20)	NSD on Nfr2 or NF-κB expressions and no antioxidant or anti-inflammatory effects.	16 wks	Stage 3-4<br><br>Non-dialysis with eGFR 15- 60 mL/min.	{Saldanha, 2016 #264}

Cereal, cookies, and bars without added fiber (C) for 2 wks,<br>(n=15)		6 wks	Stage 3-5, NI	N/A	{Salmean, 2013 #125}

then similar food with added fiber (23 g/day) for 4 wks<br>(n=15, same as prior)	↓ constipation with ↑ stool frequency, improved lipid profile, & no change in overall QOL.				
Freshly squeezed pomegranate juice (PJ) for 8 weeks, (n=41)<br><br>then washout for 4 weeks, and UC for 8 weeks (or vice versa) (n=41, same as prior)	↓ BP, triglycerides.<br>↑ HDL.<br><br>Signs of ↓ oxidative stress (e.g., ↑ total antioxidant capacity) & ↓ inflammation (e.g., ↓ IL-6).	20 wks	ESRD, NI, NI	HD ≥ 3 mo	{Barati Boldaji, 2020 #128}
Fish oil (4.6 g n-3) supplement<br>(n=14)<br><br>OO supplement<br>(n=15)	Both:<br>↑ fibrinolytic potential after 6 months. No change in transcapillary escape rate of albumin or procoagulant activity.	1 y	NI, NI, UA > 300 mg/day	Diabetic nephropathy with albuminuria. Insulin- dependent DM.	{Myrup, 2001 #135}
